# Differential properties of KRAS transversion and transition mutations in non-small cell lung cancer: associations with environmental factors and clinical outcomes

**DOI:** 10.1186/s12885-022-10246-7

**Published:** 2022-11-08

**Authors:** Koichi Sato, Hiroaki Akamatsu, Yasuhiro Koh, Koichi Ogawa, Shun-ichi Isa, Masahiko Ando, Akihiro Tamiya, Akihito Kubo, Chiyoe Kitagawa, Tomoya Kawaguchi, Nobuyuki Yamamoto

**Affiliations:** 1 Internal Medicine III, Wakayama Medical University, 811-1 Kimiidera, Wakayama-shi, Wakayama, 641-8509 Japan; 2 Center for Biomedical Sciences, Wakayama Medical University, 811-1 Kimiidera, Wakayama-shi, Wakayama, 641-8509 Japan; 3 Department of Respiratory Medicine, Osaka Metropolitan University Graduate School of Medicine, 1-4-3 Asahi-cho, Abeno-ku, Osaka-shi, Osaka, 545-8585 Japan; 4 Clinical Research Center, National Hospital Organization Kinki-Chuo Chest Medical Center, 1180 Nagasone-cho, Kita-ku, Sakai-shi, Osaka, 591-8555 Japan; 5 Department of Advanced Medicine, Nagoya University Hospital, 65 Tsurumai-cho, Showa-ku, Nagoya-shi, Aichi, 466-8560 Japan; 6 Internal Medicine, National Hospital Organization Kinki-Chuo Chest Medical Center, 1180 Nagasone-cho, Kita-ku, Sakai-shi, Osaka, 591-8555 Japan; 7 Medical Oncology, Oncology Center, Aichi Medical University School of Medicine, 1-1 Yazakokarimata, Nagakute-shi, Aichi, 480-1195 Japan; 8 Medical Oncology and Respiratory Medicine, National Hospital Organization Nagoya Medical Center, 4-1-1 Sannomaru, Naka-ku, Nagoya-shi, Aichi, 460-0001 Japan

**Keywords:** KRAS, Transversion, Transition, Smoking, Environmental factors, Non-small cell lung cancer

## Abstract

**Background:**

KRAS-mutated non-small cell lung cancer (NSCLC) accounts for 23–35% and 13–20% of all NSCLCs in white patients and East Asians, respectively, and is therefore regarded as a major therapeutic target. However, its epidemiology and clinical characteristics have not been fully elucidated because of its wide variety of mutational subtypes. Here, we focused on two distinct base substitution types: transversion mutations and transition mutations, as well as their association with environmental factors and clinical outcome.

**Methods:**

Dataset from the Japan Molecular Epidemiology Study, which is a prospective, multicenter, and molecular study epidemiology cohort study involving 957 NSCLC patients who underwent surgery, was used for this study. Questionnaire-based detailed information on clinical background and lifestyles was also used to assess their association with mutational subtypes. Somatic mutations in 72 cancer-related genes were analyzed by next-generation sequencing, and KRAS mutations were classified into three categories: transversions (G > C or G > T; G12A, G12C, G12R, G12V), transitions (G > A; G12D, G12S, G13D), and wild-type (WT). Clinical correlations between these subtypes have been investigated, and recurrence-free survival (RFS) and overall survival (OS) were evaluated.

**Results:**

Of the 957 patients, KRAS mutations were detected in 80 (8.4%). Of these, 61 were transversions and 19 were transitions mutations. Both pack-years of smoking and smoking duration had significant positive correlation with the occurrence of transversion mutations (*p* = 0.03 and < 0.01, respectively). Notably, transitions showed an inverse correlation with vegetable intake (*p* = 0.01). Patients with KRAS transitions had the shortest RFS and OS compared to KRAS transversions and WT. Multivariate analysis revealed that KRAS transitions, along with age and stage, were significant predictors of shorter RFS and OS (HR 2.15, *p* = 0.01; and HR 2.84, *p* < 0.01, respectively).

**Conclusions:**

Smoking exposure positively correlated with transversions occurrence in a dose-dependent manner. However, vegetable intake negatively correlated with transitions. Overall, KRAS transition mutations are significantly poor prognostic factors among resected NSCLC patients.

**Supplementary Information:**

The online version contains supplementary material available at 10.1186/s12885-022-10246-7.

## Background

Lung cancer is the leading cause of death from various cancers in many countries [1]. Kirsten rat sarcoma viral oncogene homolog (KRAS) is a well-known oncogene that drives cancer progression to metastases in various types of carcinomas, including non-small cell lung cancer (NSCLC), pancreatic cancer, and colorectal cancer. KRAS mutations were found in 23–35% in white patients [2–4] and in 13–20% in Asians [5, 6]. Among KRAS mutations in NSCLC, G12C was the most common (32–39%), followed by G12V (18–21%) and G12D (17–23%), G12A/G12S/G12R (16%), G13C/G13D/G13S (7%) and Q61H/Q61K (0.7%) [7, 8]. KRAS mutations have long been untreatable owing to their unique shapes. However, inhibitors of KRAS G12C, such as sotorasib, have recently been developed, and the first KRAS-targeted anticancer therapy was then made available [9]. Advances in KRAS-targeted therapies are anticipated in the future. Thus, KRAS subtypes need to be characterized. Previous studies have attempted to elucidate the characteristics of each of the KRAS mutations; however, different frequencies of each subtype mutation rendered the results inconsistent. According to the base substitutions, KRAS mutations have been categorized as transversion mutations (G > C or G > T; G12A, G12C, G12R, G12V) or transition mutations (G > A; G12D, G12S, G13D). Transitions refers to the substitution of purine with purine (adenine to guanine), or pyrimidine to pyrimidine (cytosine to thymine), whereas transversions correspond to the substitution of purine with pyrimidine [10]. Although transversions mutations are basically considered as smoking-related substitutions, [11] actual associations have not yet been completely investigated. Other clinical factors associated with the occurrence of transversions and transitions have not yet been elucidated. Additionally, structural differences between the P-loop and Switch-II were reported between transversions and transitions; however, it has not been clarified whether this leads to phenotypical outcomes [12]. Although this background information enables us to recognize two distinct subtypes of KRAS mutations, the clinical implications of these remain unclear. Here, we describe the clinical differences of KRAS mutations between transversions and transitions.

## Methods

### Study design

The Japan Molecular Epidemiology (JME) study is a prospective, multicenter, molecular epidemiology cohort study of surgically resected NSCLC patients in Japan. The study included 957 patients who underwent curative-intent surgery for clinical stage I-IIIB disease (American Joint Committee on Cancer [AJCC] version 7) [13–15]. The patients were required to complete the questionnaire before surgery, which included questions on lifestyle factors (smoking status, body mass index (BMI), exercise, high fat diet, vegetables, fruits, and soybean intake). This questionnaire was designed for the SWOG S0424 study [16]. Pack-years of smoking were between 0 ≤ 30, 30 ≤ 60, or 60 + pack years. Smoking duration was categorized as, 0 ≤ 20, 20 ≤ 40, or 40 + years. Fruits and vegetable intake was categorized as zero, 1–2, 3–4, 5 + servings per week. Other detailed eligibility criteria and questionnaire have been previously reported elsewhere [14]. Resected specimens were analyzed for 72 cancer-associated somatic mutations (*ABL1, CSF1R, FGFR3, JAK2, NOTCH1, RET, AKT1, CTNNB1, FLT3, JAK3, NPM1, SMAD4, ALK, epidermal growth factor receptor [EGFR], GNA11, KDR, NRAS, SMARCB1, APC, ERBB2, GNAQ, KIT, PDGFRA, SMO, ATM, ERBB4, GNAS, KRAS, PIK3CA, SRC, BRAF, FBXW7, HNF1A, MET, PTEN, STK11, CDH1, FGFR1, HRAS, MLH1, PTPN11, TP53, CDKN2a, FGFR2, IDH1, MPL, RB1, VHL, NF1, SMARC4, KEAP1, ARID1A, RBM10, SETD1, CBL, CUL3, DDR2, RASA1, TSC1, TSC2, CTIF, ERBB3, NFE2L2, PPP2R1A, AKT3, BRD3, CCND1, MYC, PTCH1, FGFR4, U2AF1, MAP2K1*) with multiplex targeted deep sequencing on MiSeq by a TruSeq Amplicon Cancer Panel and an additional custom panel (Illumina, San Diego, California, USA). Regarding KRAS mutations, recurrence-free survival (RFS) and overall survival (OS) were evaluated and compared among patients with KRAS wild-type (WT), transversions, and transitions mutations. The time period for checking for recurrence by imaging follow-up was not specified.

### Statistical analysis

The clinical backgrounds of patients with KRAS WT, transversions, and transitions mutations were compared using Fisher’s exact test. Dose-dependency within each item was assessed using the least-squares method. To analyze prognosis, RFS and OS were assessed using the Kaplan–Meier method. Cox proportional hazards models were used for the determination of adjusted hazard ratios (HR) and 95% confidence intervals (CI). Univariate and multivariate logistic regression models by Cox proportional hazards model were used to explore prognostic factors for RFS and OS survival. The factors for continuous variables were dichotomized using the median or between ≤ 2/ weeks and ≥ 3/ weeks. Multivariate analysis of RFS and OS was performed for these significant univariate factors. A statistical *p*-value less than 0.05 indicated significance. Statistical analysis was conducted using JMP version 14 (SAS Institute Inc, USA).

## Results

### Clinical background

Patients were enrolled between July 2012 and December 2013, and followed up for at least four years. Nine hundred and fifty-seven patients were enrolled from 43 institutions in Japan. Of those, 876 cases (91.5%) were successfully analyzed for genetic mutations. After the exclusion of two patients with co-mutations (*KRAS* and *EGFR*), 80 patients had KRAS mutations, which comprised 61 transversion (G12C: 26, G12V: 19, G12A: 14 and G12R: 2) and 19 transition (G12D: 16, G13D: 2 and G12S: 1) mutations, respectively.

**Fig. 1:**
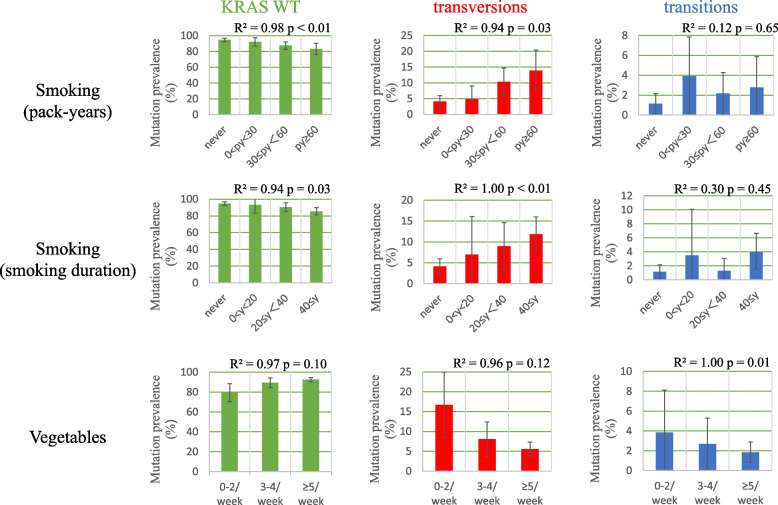
Lifestyle factors assessed in a dose-dependent manner using the least squares method. *R*^2^ = coefficient of determination. KRAS WT, Kirsten rat sarcoma viral oncogene homolog wild-type; py, pack-years; y, years

Table 1 shows the clinical characteristics of each group. Compared with KRAS WT, both transversions and transitions were significantly more common in men, ever-smokers, and non-squamous cell carcinoma patients. In addition, transitions had significantly more co-mutations than KRAS WT. Among the transitions, 57.9% had concurrent mutations other than KRAS mutations. Common co-mutations with transversions were TP53 (24.6%) and PIK3CA (13.1%), while PIK3CA (21.1%) and TP53 (15.8%) were common with transitions. The KRAS transversions group had a higher BMI and less frequent intake of fruits and vegetables than KRAS WT. To understand the influence of these backgrounds, we explored the dose-dependent correlation between KRAS subgroups and lifestyle items. KRAS transversions showed a significant correlation with pack-years of smoking (*R*^2^ = 0.94, *p* = 0.03), whereas KRAS WT was inversely correlated (*R*^2^ = 0.98, *p* < 0.01). Smoking duration also showed a significant positive correlation with transversions (*R*^2^ = 1.00, *p* < 0.01) and KRAS WT was inversely correlated (*R*^2^ = 0.94, *p* = 0.03). Among the transitions, only vegetable intake had a significant negative dose-dependency (R^2^ = 1.00, *p* = 0.01) (Fig. 1). Correlations between other clinical factors and KRAS subgroups are described in Supplementary Fig. 1.

**Table 1:** Clinical and lifestyle background

	**KRAS WT (*n* = 794) No. (%)**	**Transversions** **(*n* = 61) No. (%)**	*P* value^*^	**Transitions (*n* = 19) No. (%)**	***P* value**^**^**^	***P* value**^**#**^
Age median (range)	70.0 (23–92)	70.0 (49–85)	-	70.0 (52–86)	-	-
Sex			0.02		< 0.01	0.65
Male	366 (46.1)	39 (63.9)		13 (68.4)		
Female	428 (53.9)	22 (36.1)		6 (31.6)		
Smoking			< 0.01		< 0.01	0.64
Never	413 (52.0)	18 (29.5)		5 (26.3)		
Ever	381 (48.0)	43 (70.5)		14 (73.7)		
Histology			0.01		0.01	1.00
Sq	138 (17.4)	3 (4.9)		1 (5.3)		
Non-sq	656 (82.6)	58 (95.1)		18 (94.7)		
Stage			0.30		0.84	0.77
I	568 (71.5)	38 (62.3)		12 (63.1)		
II	114 (14.4)	12 (19.7)		3 (15.8)		
III	94 (11.8)	7 (11.5)		3 (15.8)		
IV	18 (2.3)	4 (6.5)		1 (5.3)		
No. of Mutations			0.35		< 0.01	0.04
0	200 (25.2)	0		0		
1	384 (48.4)	35 (57.4)		8 (42.1)		
≥ 2	210 (26.4)	26 (42.6)		11 (57.9)		
BMI			0.01		0.06	0.39
< 22.5	402 (50.6)	20 (32.8)		7 (36.9)		
≥ 22.5	389 (49.0)	40 (65.6)		12 (63.1)		
Unknown	3 (0.4)	1 (1.6)		0		
High fat diets			0.12		0.32	0.67
≤ 2/ week	313 (39.4)	31 (50.8)		9 (47.4)		
≥ 3/ week	477 (60.1)	30 (49.2)		10 (52.6)		
Unknown	4 (0.5)	0		0		
Vegetables			0.01		0.13	0.47
≤ 2/ week	62 (7.8)	13 (21.3)		3 (15.8)		
≥ 3/ week	732 (92.2)	48 (78.7)		16 (84.2)		
Fruits			0.02		0.14	0.48
≤ 2/ week	249 (31.4)	29 (47.5)		8 (42.1)		
≥ 3/ week	545 (68.6)	32 (52.5)		11 (57.9)		
Soy bean			0.05		0.84	0.12
≤ 2/ week	112 (14.1)	16 (26.2)		3 (15.8)		
≥ 3/ week	681 (85.8)	45 (73.8)		16 (84.2)		
Unknown	1 (0.1)	0		0		
Exercise			1.00		0.15	0.15
≤ 2/ week	409 (51.5)	32 (52.5)		12 (63.1)		
≥ 3/ week	385 (48.5)	29 (47.5)		7 (36.9)		

Abbreviations: *KRAS WT* Kirsten rat sarcoma viral oncogene homolog wild-type, *Sq* squamous cell carcinoma, *BMI* body mass index

^*^KRAS WT vs. transversions

^^^KRAS WT vs. transitions

^#^transversions vs. transitions

### Prognosis

**Fig. 2:**
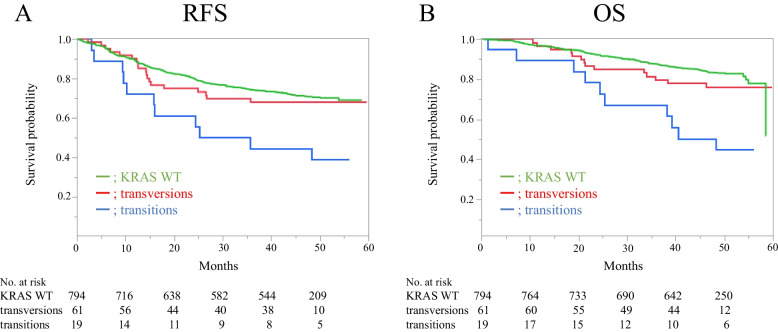
Kaplan–Meier curves of KRAS WT, transversions, and transitions for (**A**) RFS and (**B**) OS, (**A**) RFS: *p* < 0.01, (**B**) OS: *p* < 0.01, log-rank test. KRAS WT, Kirsten rat sarcoma viral oncogene. homolog wild-type; RFS, recurrence-free survival; OS, overall survival

Figure 2 shows the RFS and OS rates of each group. The median RFS of patients with KRAS transitions was 30.4 months, which was the shortest compared to KRAS WT and KRAS transversions, and their medians were not reached. Compared with transitions, HR between WT and transitions was 0.39 (95% CI: 0.21–0.71, *p* < 0.01), while that between transversions and transitions was 0.44 (95% CI: 0.21–0.93, *p* = 0.03) (Fig. 2A). The OS in each subgroup showed a similar trend. Median OS with transitions was 48.3 months, and KRAS WT and transversions OS medians did not reach. HR between WT and transitions was 0.26 (95% CI: 0.14–0.50, *p* < 0.01), while that between transversions and transitions was 0.36 (95% CI: 0.16–0.82, *p* = 0.02) (Fig. 2B).

Univariate analysis for RFS showed that KRAS transitions, age (< 70 years), male sex, never smoker, squamous histology, stage I, and low intake of high-fat diet were significant prognostic factors. Multivariate analysis revealed that transitions, age (≥ 70 years), and stage (≥ II) were significantly poor prognostic factors (Table 2). OS, transitions, age (< 70 years), male sex, never smoked, squamous histology, stage I, high-fat diet (≤ 2 per week), vegetables (≤ 2 per week) were significant prognostic factors in the univariate analysis. Multivariate test showed that transitions, age (≥ 70 years), and stage (≥ II) were significantly poor OS factors (Table 3). Co-mutations with KRAS transversions and transitions did not impact prognosis.

**Table 2:** Univariate and multivariate analysis for RFS

RFS	Univariate analysis	Multivariate analysis
transversions vs. the others	HR 1.10 (95% CI: 0.69–1.75),	-
(KRAS WT and transitions)	*p* = 0.71	
transitions vs. the others	HR 2.53 (95% CI: 1.38–4.63),	HR 2.15 (95% CI: 1.17–3.97),
(KRAS WT and transversions)	*p* < 0.01	*p* = 0.01
Age:	HR 0.36 (95% CI: 0.16–0.84),	HR 0.64 (95% CI: 0.49–0.83),
< 70 vs. ≥ 70	*p* < 0.01	*p* < 0.01
Sex:	HR 1.61 (95% CI: 1.26–2.06),	HR 1.29 (95% CI: 0.90–1.87),
Male vs. Female	*p* < 0.01	*p* = 0.17
Smoking:	HR 0.60 (95% CI: 0.47–0.77),	HR 0.91 (95% CI: 0.62–1.33),
Never vs. current / ever	*p* < 0.01	*p* = 0.62
Histology:	HR 1.51 (95% CI: 1.12–2.04),	HR 0.90 (95% CI: 0.64–1.27),
Sq vs. non-sq	*p* < 0.01	*p* = 0.55
Stage:	HR 0.22 (95% CI: 0.17–0.29),	HR 0.23 (95% CI: 0.18–0.30),
I vs. (II, III, IV)	*p* = 0.01	*p* < 0.01
Mutations:	HR 0.97 (95% CI: 0.73–1.30),	-
0 vs. ≥ 1	*p* = 0.81	
BMI:	HR 0.79 (95% CI: 0.62–1.01),	-
< 22.5 vs. ≥ 22.5	*p* = 0.06	
High fat diet:	HR 1.41 (95% CI: 1.10–1.80),	HR 1.15 (95% CI: 0.89–1.49),
≤ 2/ week vs. ≥ 3/ week	*p* < 0.01	*p* = 0.27
Vegetables:	HR 1.33 (95% CI: 0.89–1.98),	-
≤ 2/ week vs. ≥ 3/ week	*p* = 0.16	
Fruits:	HR 0.99 (95% CI: 0.76–1.28),	-
≤ 2/ week vs. ≥ 3/ week	*p* = 0.94	
Soy bean:	HR 1.33 (95% CI: 0.97–1.82),	-
≤ 2/ week vs. ≥ 3/ week	*p* = 0.08	
Exercise:	HR 0.98 (95% CI: 0.77–1.26),	-
≤ 2/ week vs. ≥ 3/ week	*p* = 0.85	

Abbreviations: *RFS* recurrence-free survival, *KRAS WT* Kirsten rat sarcoma viral oncogene homolog wild-type, *Sq* squamous cell carcinoma. Carcinoma, *BMI* body mass index, *HR* hazard ratio, *95% CI* 95% confidence interval

**Table 3:** Univariate and multivariate analysis for OS

OS	Univariate analysis	Multivariate analysis
transversions vs. the others	HR 1.33 (95% CI: 0.77–2.31),	-
(KRAS WT and transitions)	*p* = 0.33	
transitions vs. the others	HR 3.74 (95% CI: 1.97–7.10),	HR 2.84 (95% CI: 1.47–5.51),
(KRAS WT and transversions)	*p* < 0.01	*p* < 0.01
Age:	HR 0.51 (95% CI: 0.36–0.70),	HR 0.52 (95% CI: 0.37–0.74),
< 70 vs. ≥ 70	*p* < 0.01	*p* < 0.01
Sex:	HR 2.15 (95% CI: 1.55–2.97),	HR 1.19 (95% CI: 0.73–1.96),
Male vs. Female	*p* < 0.01	*p* = 0.49
Smoking:	HR 0.39 (95% CI: 0.28–0.55),	HR 0.64 (95% CI: 0.38–1.09),
Never vs. current / ever	*p* < 0.01	*p* = 0.10
Histology:	HR 2.44 (95% CI: 1.73–3.44),	HR 1.45 (95% CI: 0.97–2.15),
Sq vs. non-sq	*p* < 0.01	*p* = 0.07
Stage:	HR 0.25 (95% CI: 0.18–0.34),	HR 0.29 (95% CI: 0.21–0.40),
I vs. (II, III, IV)	*p* < 0.01	*p* < 0.01
Mutations:	HR 0.78 (95% CI: 0.55–1.12),	-
0 vs. ≥ 1	*p* = 0.17	
BMI:	HR 0.84 (95% CI: 0.61–1.15),	-
< 22.5 vs. ≥ 22.5	*p* = 0.27	
High fat diet:	HR 1.63 (95% CI: 1.19–2.23),	HR 1.23 (95% CI: 0.89–1.71),
≤ 2/ week vs. ≥ 3/ week	*p* < 0.01	*p* = 0.21
Vegetables:	HR 1.82 (95% CI: 1.15–2.89),	HR 1.63 (95% CI: 1.00–2.65),
≤ 2/ week vs. ≥ 3/ week	*p* = 0.02	*p* = 0.05
Fruits:	HR 1.14 (95% CI: 0.82–1.58),	-
≤ 2/ week vs. ≥ 3/ week	*p* = 0.44	
Soy bean:	HR 1.36 (95% CI: 0.92–2.03),	-
≤ 2/ week vs. ≥ 3/ week	*p* = 0.14	
Exercise:	HR 0.99 (95% CI: 0.73–1.35),	-
≤ 2/ week vs. ≥ 3/ week	*p* = 0.95	

Abbreviations: *OS* overall survival, *KRAS WT* Kirsten rat sarcoma viral oncogene homolog wild-type, *Sq* squamous cell carcinoma, *BMI* body mass index, *HR* hazard ratio, *95% CI* 95% confidence interval

## Discussion

Thirty years since KRAS mutations have been recognized as oncogenes, several anticancer drugs targeting KRAS have also been identified [17]. Recently, sotorasib, a first-in-class KRAS G12C inhibitor, demonstrated promising results in the CodeBreaK100 clinical phase 2 trial. In this trial, the objective response rate was 37.1% and the median progression-free survival (PFS) was 6.8 months [18]. Adagrasib is another promising KRAS G12C inhibitor that objective response rate was 42.9% and the median progression-free survival was 6.5 months in the phase 2 cohort of the KRYSTAL-1 trial [19]. Other agents targeting KRAS mutations are under investigation in early phase trials. Thus, subtyping KRAS mutations has become critical in clinical practice. Treatment for mutations other than KRAS G12C is expected to be available in the future, with each mutation needing to be characterized. Previous reports revealed that smokers are likely to harbor KRAS mutations [20]. However, detailed clinical characteristics for each subtype have not been elucidated. Additionally, fractionating their subtypes was hampered in outlining their clinical and prognostic differences. In this study, based on substitutions, we categorized KRAS mutations into two types (transversions and transitions), and investigated their clinical relevance.

Importantly, using the detailed questionaries, we revealed the clinical differences between KRAS transversions and transitions not only with qualitative lifestyle information, but with quantitative exposures. Regarding smoking, only transversions showed a significant positive correlation with smoking exposure (pack-years and duration) in a dose-dependent manner. KRAS transversions (G > T) in patients with lung cancer is thought to be caused by exposure to polycyclic aromatic hydrocarbons, such as benzopyrene in cigarettes [21]. Thus, the more one smokes, the more transversions one is likely to have. However, this study showed that the frequency of KRAS transitions had significantly negative correlation with vegetable intake. Vegetables contain flavanols, which can inhibit the formation of nitroso compounds. Nitroso compounds induce alkylation of guanine bases, which could have caused transitions [22, 23]. It has also been reported that lupeol, a substance abundant in vegetables, suppresses STAT3 activation, [24] which is upregulated in KRAS mutant lung cancer [25]. It is possible that a lack of protective vegetable intake could predispose patients to G12D mutation (transition mutation). Therefore, G12D may have a greater involvement of aberrant STAT3, which is different from the RAS pathway [26]. To the best of our knowledge, this study is the first to distinguish the clinical backgrounds of KRAS transversions and transitions, and the correlation between transitions and lower vegetable intake in lung cancer patients. Interestingly, transitions had a higher percentage of co-occurring mutations compared to KRAS WT and transversions (Table 1).  As observed in EGFR-mutated NSCLC, co-mutation may have a negative prognostic role [27]. However, owing to the small sample size, the prognostic value of the number of co-mutations with KRAS was not observed in this study. Patients with KRAS transitions had significantly shorter RFS and OS than KRAS WT or transversions. In addition, multivariate analysis demonstrated that KRAS transitions was a negative prognostic factor for both RFS and OS. Among resected NSCLC, Finn et al. reported that G12C, a subtype of transversions, had a significantly shorter OS than the other KRAS mutations, [28] whereas another report showed the opposite result [29]. These differences in prognosis can be attributed that the KRAS isoforms are highly heterogeneous. They correlate with different therapeutic responses to MEK inhibitors, with the KRAS G12C and Q61H variants being more susceptible than the other isoforms. The authors also reported that in patients with NSCLC who underwent comprehensive tumor genome profiling, STK11 and ATM mutations were significantly enriched in tumors harboring G12C, G12A and G12V. KEAP1 were significantly enriched in G12C and G13X [30]. Thus, even considering G12C, one of the main transversions, there are differences by isoforms and co-mutations in each subtype that could impact the prognostic differences between KRAS transversions and transitions.

The strength of our study was that it demonstrates that KRAS transitions was an independent poor prognostic factor via multivariate analysis. Preclinical evidence supports this prognostic difference which may be attributable to the difference in signal cascades. G12D, a subtype of KRAS transitions, is associated with phosphorylation in vitro and in vivo, thus activating PI3K/AKT and MEK cascades, whereas KRAS transversion mutations, such as G12V and G12C, activate the RalGDS pathway and decrease phosphorylation of AKT [31, 32]. Our result highlighted the clinical differences in KRAS subtypes based on base substitutions. Thus, a similar approach can be applied to other rare mutations.

A limitation of this study was that the objective cases included only postoperative patients who were exclusively Japanese. Further, the number of patients in each KRAS subgroup was limited, especially those in the transition mutation group. Therefore, we were not able to investigate the differences in co-mutation characteristics between the subtypes. There were also mutations that could not be ascertained by only this next generation sequencing panel. This panel covers almost 15% of cancer-associated mutations which occurred in various malignancies. Future studies with large-scale KRAS-positive cases are needed. The cases in this study were before the advent of immunotherapies. Therefore, introduction of immune-checkpoint inhibitor in the perioperative setting may alter the prognosis of KRAS-mutated NSCLC.

## Conclusions

Smoking exposure positively correlated with KRAS transversions occurrence in a dose-dependent manner. However, vegetable intake negatively correlated with KRAS transitions. KRAS transitions were found to be a significant poor prognostic factor among patients with resected NSCLC.

## Supplementary Information

**Additional file 1: Supplemental Figure 1. **Lifestyle factors assessed in a dose-dependent manner using the least-squares method.

## Data Availability

The datasets used and/or analyzed during the current study are available from the corresponding author upon reasonable request.

## Abbreviations

KRAS: Kirsten rat sarcoma viral oncogene homolog
NSCLC: Non-small cell lung cancer
RFS: Recurrence-free survival
OS: Overall survival
WT: Wild-type
HR: Hazard ratios
CI: Confidence interval
EGFR: Epidermal Growth Factor Receptor
PFS: Progression-free survival
