# Supplementary material for: Differential properties of KRAS transversion and transition mutations in non-small cell lung cancer: associations with environmental factors and clinical outcomes
Source: BMC Cancer. 2022 Nov 8;22:1148. doi: 10.1186/s12885-022-10246-7 (PMC9641926; doi:10.1186/s12885-022-10246-7)
Supplement: Supplementary file 1 — Additional file 1: Supplemental Figure 1. Lifestyle factors assessed in a dose-dependent manner using the least-squares method. [file 12885_2022_10246_MOESM1_ESM.pptx]

## Slide 1
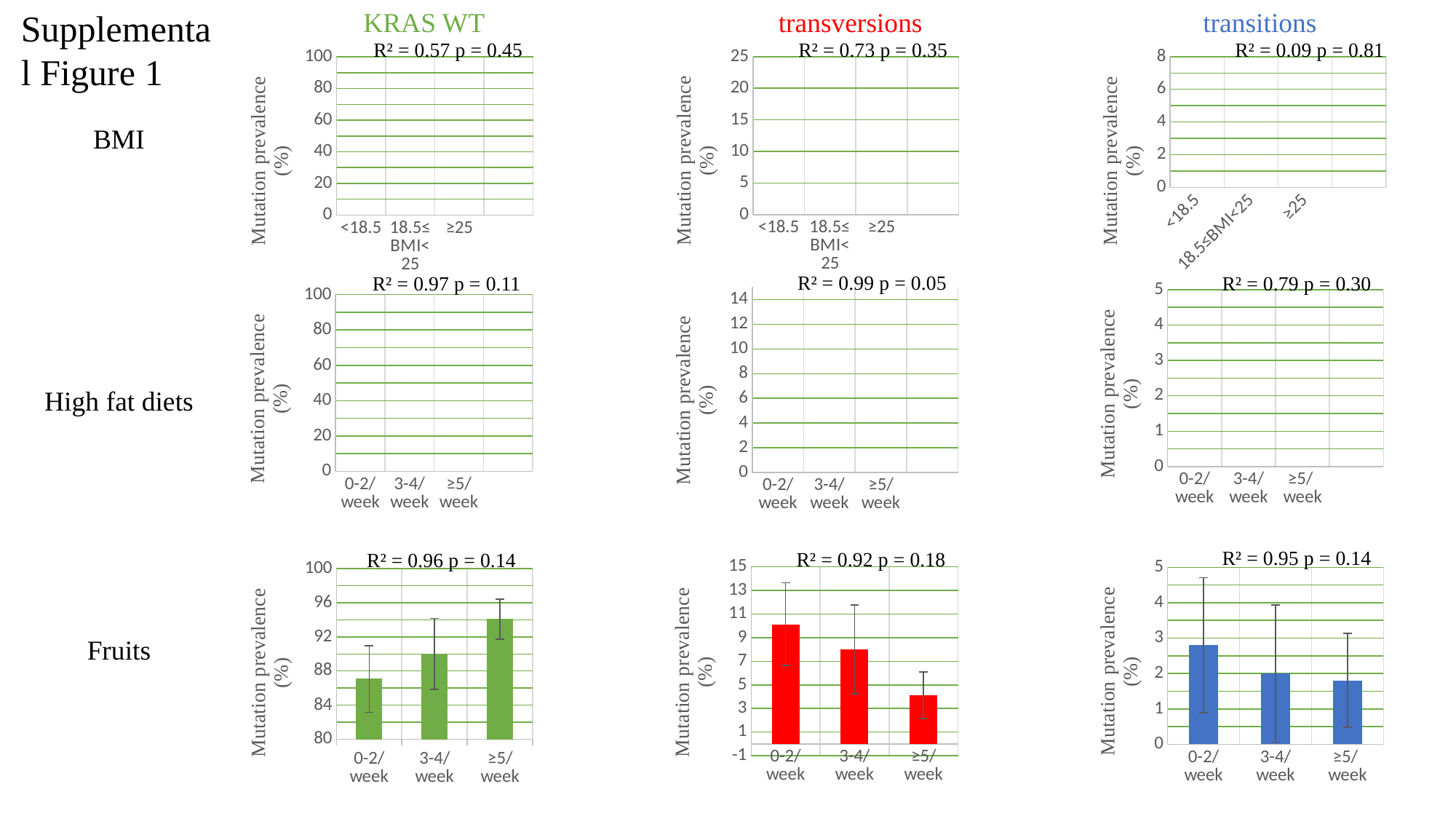

Supplemental Figure 1
transversions
KRAS WT
transitions
R² = 0.57 p = 0.45
R² = 0.73 p = 0.35
R² = 0.09 p = 0.81
### Chart
| Category | KRAS G12D G12S G13D |
|---|---|
| <18.5 | 6.349 |
| 18.5≤BMI<25 | 5.314 |
| ≥25 | 12.9 |
### Chart
| Category | KRAS G12D G12S G13D |
|---|---|
| <18.5 | 3.175 |
| 18.5≤BMI<25 | 1.932 |
| ≥25 | 2.688 |
### Chart
| Category | KRAS G12D G12S G13D |
|---|---|
| <18.5 | 90.5 |
| 18.5≤BMI<25 | 92.8 |
| ≥25 | 84.9 |BMI
R² = 0.99 p = 0.05
R² = 0.97 p = 0.11
R² = 0.79 p = 0.30
### Chart
| Category | KRAS G12D G12S G13D |
|---|---|
| 0-2/ week | 2.55 |
| 3-4/ week | 2.37 |
| ≥5/
week | 1.52 |
### Chart
| Category | KRAS G12D G12S G13D |
|---|---|
| 0-2/ week | 88.7 |
| 3-4/ week | 90.9 |
| ≥5/ week | 93.6 |
### Chart
| Category | KRAS G12D G12S G13D |
|---|---|
| 0-2/ week | 8.78 |
| 3-4/ week | 6.72 |
| ≥5/ week | 4.92 |High fat diets
R² = 0.95 p = 0.14
R² = 0.92 p = 0.18
R² = 0.96 p = 0.14
### Chart
| Category | KRAS G12D G12S G13D |
|---|---|
| 0-2/ week | 2.8 |
| 3-4/ week | 2.0 |
| ≥5/
week | 1.8 |
### Chart
| Category | KRAS G12D G12S G13D |
|---|---|
| 0-2/ week | 10.1 |
| 3-4/ week | 8.0 |
| ≥5/ week | 4.12 |
### Chart
| Category | KRAS G12D G12S G13D |
|---|---|
| 0-2/ week | 87.1 |
| 3-4/ week | 90.0 |
| ≥5/ week | 94.1 |
### Chart
| Category |
|---|
### Chart
| Category |
|---|Fruits

## Slide 2
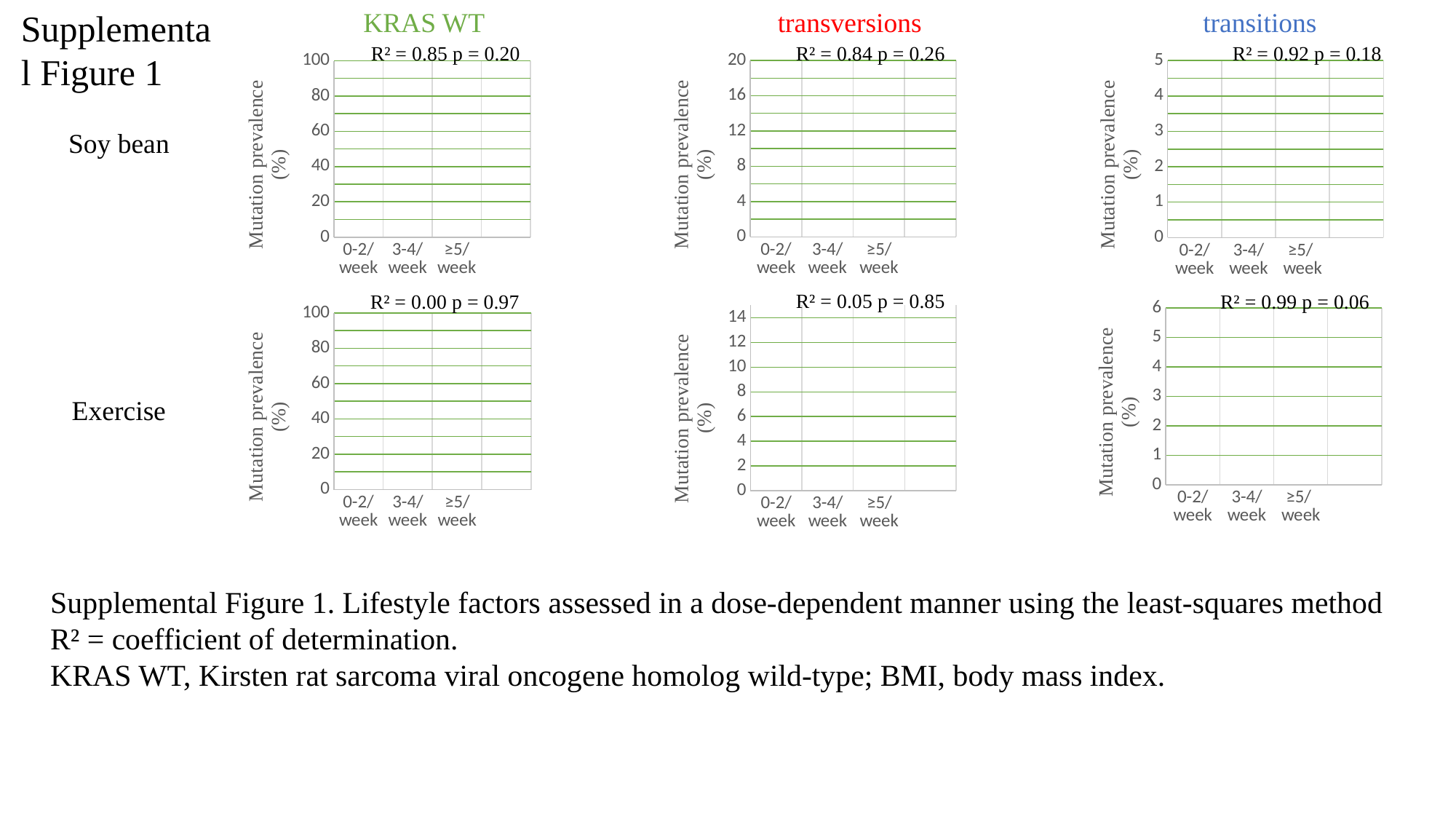

Supplemental Figure 1
KRAS WT
transversions
transitions
R² = 0.85 p = 0.20
R² = 0.84 p = 0.26
R² = 0.92 p = 0.18
### Chart
| Category | KRAS G12D G12S G13D |
|---|---|
| 0-2/ week | 12.2 |
| 3-4/ week | 10.3 |
| ≥5/ week | 4.26 |
### Chart
| Category | KRAS G12D G12S G13D |
|---|---|
| 0-2/ week | 2.29 |
| 3-4/ week | 2.23 |
| ≥5/
week | 2.12 |
### Chart
| Category | KRAS G12D G12S G13D |
|---|---|
| 0-2/ week | 85.5 |
| 3-4/ week | 87.5 |
| ≥5/ week | 93.6 |Soy bean
R² = 0.05 p = 0.85
R² = 0.00 p = 0.97
R² = 0.99 p = 0.06
### Chart
| Category | KRAS G12D G12S G13D |
|---|---|
| 0-2/ week | 2.65 |
| 3-4/ week | 1.89 |
| ≥5/
week | 1.53 |
### Chart
| Category | KRAS G12D G12S G13D |
|---|---|
| 0-2/ week | 90.3 |
| 3-4/ week | 95.0 |
| ≥5/ week | 89.3 |
### Chart
| Category | KRAS G12D G12S G13D |
|---|---|
| 0-2/ week | 7.06 |
| 3-4/ week | 3.14 |
| ≥5/ week | 9.16 |Exercise
Supplemental Figure 1. Lifestyle factors assessed in a dose-dependent manner using the least-squares method
R² = coefficient of determination.
KRAS WT, Kirsten rat sarcoma viral oncogene homolog wild-type; BMI, body mass index.
### Chart
| Category |
|---|
### Chart
| Category |
|---|
